# Überblick zu chronobiologischen und schlafmedizinischen Aspekten bei Depressionen im Jugendalter

**DOI:** 10.1007/s00103-024-03853-1

**Published:** 2024-03-12

**Authors:** Neda Ghotbi, Aline Doreen Scherff, Ellen Greimel, Gerd Schulte-Körne

**Affiliations:** Kinder- und Jugendpsychiatrie, Klinik und Poliklinik für Kinder- und Jugendpsychiatrie, Psychosomatik und Psychotherapie, Nussbaumstr. 5a, 80336 München, Deutschland

**Keywords:** Chronotyp, Sozialer Jetlag, Jugendliche, Schlafstörung, Insomnie, Chronotype, Social jetlag, Youth, Sleep disorder, Insomnia

## Abstract

Bei Jugendlichen mit Depression werden mit einer Häufigkeit von bis zu 71 % Veränderungen des Schlafes berichtet. In dieser narrativen Übersichtsarbeit werden chronobiologische und schlafmedizinische Aspekte bei Depressionen im Jugendalter basierend auf der aktuellen Forschungsliteratur zusammenfassend dargestellt. Die circadiane Uhr des Menschen ermöglicht die Synchronisierung unseres Organismus mit der Licht-Dunkel-Struktur der Umwelt. Die individuelle Synchronisierung wird als Chronotyp bezeichnet. Der Chronotyp verändert sich u. a. altersabhängig und Jugendliche weisen die spätesten Chronotypen auf. Wenn es durch frühe Schul‑/Arbeitszeiten zu einer Diskrepanz zwischen Chronotyp und Außenzeit kommt, entsteht der sog. soziale Jetlag. Dieser soziale Jetlag tritt im Jugendalter vermehrt auf und ist mit zahlreichen gesundheitlichen Risiken, wie zum Beispiel Depressionen, verbunden. Veränderungen des Schlafes sind im Zusammenhang mit Depressionen gut beschrieben und treten im hohen Maße komorbid zu affektiven Erkrankungen auf. In diesem Artikel werden zu Beginn grundlegende Konzepte der Chronobiologie und schlafmedizinischer Aspekte zusammengefasst. Anschließend werden Gesundheitsrisiken und Zusammenhänge zu Depressionen spezifisch für Jugendliche erläutert, bevor dieser Artikel mit Empfehlungen für die klinische Versorgung bei Schlafstörungen und Depressionen im Jugendalter sowie für weitere Forschungsvorhaben schließt.

## Abstract (en)

Changes in sleep are reported in adolescents with depression with a frequency of up to 71%. Aspects of chronobiology and sleep based on the current scientific literature are illustrated and summarized in this narrative review. The circadian clock synchronizes organisms to the light–dark structure of the environment. The individual synchronization is called “chronotype.” Chronotype changes according to age, among other factors, and adolescents experience the latest chronotypes overall. The potential discrepancy between internal and external time is called “social jetlag.” Social jetlag is especially pronounced during adolescence. It is associated with numerous health risks, such as depression. Changes in sleep behavior in affective disorders and its comorbidity to depression have also been well described in the literature. In this article, underlying concepts from chronobiology and sleep medicine are initially summarized. Then, health risks of disrupted sleep–wake behavior are described, and connections to depression specifically during adolescence are drawn. The article concludes with clinical recommendations for sleep disorders and depression during adolescence as well as suggestions for further research.

## Einleitung

Erkenntnisse zur Relevanz von Schlaf und der circadianen Uhr für die psychische Gesundheit von Jugendlichen nehmen fortlaufend zu. Insbesondere bestehen zahlreiche Untersuchungen zum Zusammenhang zwischen Schlaf, der circadianen Uhr und affektiven Erkrankungen. Diese unterstreichen die Bedeutung eines verbesserten Verständnisses von schlafmedizinischen und chronobiologischen Prozessen innerhalb der psychiatrisch-psychologischen Versorgung von Kindern und Jugendlichen [1].

In dieser narrativen Übersichtsarbeit wird ein Überblick zur Rolle von Schlaf und der circadianen Uhr bei depressiven Erkrankungen im Jugendalter gegeben. Es erfolgt zunächst eine kurze Einführung in grundlegende Konzepte. Anschließend werden gesundheitliche Risiken bei Abweichungen der inneren und äußeren Uhr sowie bei verändertem Schlaf aufgezeigt und hierbei wird insbesondere auf Zusammenhänge zur Depression hingewiesen. Chrono- und schlafmedizinische Untersuchungsmethoden sowie entsprechende Behandlungsmöglichkeiten werden mit dem Fokus auf Jugendliche beleuchtet und abschließend werden Handlungsempfehlungen für den wissenschaftlichen und klinischen Bereich gegeben.

### Circadiane Uhr, Chronotyp und sozialer Jetlag.

**Figure 1:**
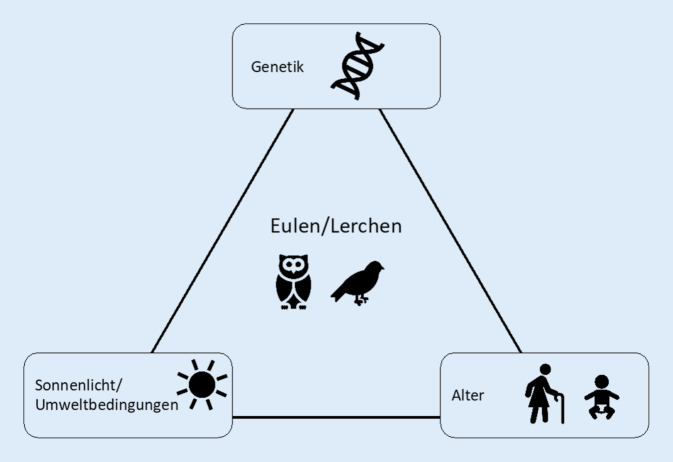

Die circadiane Uhr ermöglicht die Synchronisierung des individuellen Organismus mit der Licht-Dunkel-Struktur der Umwelt. Nicht jede Synchronisierung zwischen Organismus und Umwelt ist gleich und es entstehen individuelle Phasenbeziehungen, sog. Chronotypen. Diese können zum Beispiel mittels der Schlafzeitpunkte oder der Melatoninausschüttung ermittelt werden. Der Chronotyp variiert aufgrund genetischer Varianz, bedingungs-, geschlechts- und altersabhängig [2]. Seine Verteilung reicht von extrem frühen Chronotypen (umgangssprachlich „Lerchen“) zu extrem späten Chronotypen („Eulen“; Abb. 1).

Der moderne Lebensstil stellt eine Herausforderung für die Synchronisierung von Innenzeit (circadianes System, „innere Uhr“) und Außenzeit (natürlicher Tag-Nacht-Rhythmus der Umwelt) dar, da beispielsweise der Tag vorwiegend in geschlossenen Räumen verbracht und abends künstliches Licht verwendet wird. Es entsteht eine zunehmende Diskrepanz zwischen der Innen- und der Außenzeit, der *soziale Jetlag*, der auch beispielsweise darin Ausdruck findet, dass die meisten Menschen einen Wecker an Arbeitstagen nutzen [3]. Jugendliche weisen u. a. entwicklungsbedingt die spätesten Chronotypen auf und der soziale Jetlag ist in diesem Alter am ausgeprägtesten [4].

### Einführung Schlaf und Schlafstörungen.

Pathologische Veränderungen des Nachtschlafs sind in Bezug auf Depressionen bei Erwachsenen und auf Basis von klinischen wie epidemiologischen Studien gut charakterisiert. Bei Jugendlichen mit einer Depression werden mit einer Häufigkeit von bis zu 71 % Veränderungen des Schlafes berichtet [5]. Typische Symptome sind eine verlängerte Einschlaflatenz, Durchschlafstörungen, vorzeitiges Aufwachen, ohne wieder in den Schlaf zu finden, sowie eine zu lange Schlafdauer. Im weiteren Sinne werden als Schlafprobleme oft auch die circadiane Rhythmusstörung (Tag-Nacht-Zyklus, soziale Taktgeber) und die damit assoziierte Tagessymptomatik erfasst, welche eine fehlende Konzentrationsfähigkeit, das Gefühl der Erschöpfung und Lethargie, fehlende Energie, Schläfrigkeit und den Tagesschlaf (Nickerchen) beinhalten [6].

## Gesundheitliche Risiken: Sozialer Jetlag und Schlafstörungen

### Gesundheitsrisiko sozialer Jetlag.

Zahlreiche Gesundheitsrisiken sind bei Erwachsenen mit dem Vorliegen von chronischem sozialen Jetlag assoziiert: Beispielsweise sind erhöhte Risiken für die Entstehung von psychischen Erkrankungen, Stoffwechselerkrankungen, Übergewicht und kardiovaskuläre Erkrankungen beschrieben [7–9]. Im Jugendalter ist das Vorliegen von sozialem Jetlag und Schlafmangel ebenfalls mit einer Reihe von Beeinträchtigungen verbunden: u. a. mit schlechteren schulischen Leistungen, verminderten kognitiven Fertigkeiten, dem Risiko für Übergewicht und Asthma [10, 11]. Ebenso sind Depressionen und schwerwiegende affektive Symptome, wie z. B. Suizidalität oder eingeschränkte Emotionsregulation, in Zusammenhang mit sozialem Jetlag und Schlafmangel beschrieben [12].

### Sozialer Jetlag und Depression.

Hinsichtlich depressiver Erkrankungen werden neben der beschriebenen Assoziation auch kausale Zusammenhänge zwischen sozialem Jetlag und Depressionen diskutiert. So werden beispielsweise bei späten Chronotypen (oder in Wintermonaten) mangelnde Lichtexposition am Tag oder weniger soziale Kontakte durch weniger Aktivität untertags beschrieben. Eine geringe Lichtexposition, die durch einen späteren Tagesbeginn/kürzere Photoperiode im Winter bedingt sein kann, führt möglicherweise dazu, dass der Chronotyp (noch) später wird und sozialer Jetlag so zunimmt. Ebenso kann ein späterer Tagesbeginn zu geringerer Teilhabe an sozialen Aktivitäten führen, was depressionsfördernd sein kann [13]. Darüber hinaus bestehen einige Hinweise dafür, dass Schlafstörungen, insbesondere bei Jugendlichen, nicht als Symptome der Depression, sondern umgekehrt die Depression als Symptom von Schlafstörungen verstanden werden kann [14]. Spezifisch bei Jugendlichen wird in der Literatur neben dem ausgeprägten sozialen Jetlag und Schlafmangel auf einen verminderten homöostatischen Schlafdruck hingewiesen, der mit dem Abbau synaptischer Nervenzellverbindungen (Pruning) assoziiert ist. So weisen Jugendliche einen weniger rasch ansteigenden Schlafdruck in Wachheitsphasen auf, während der Chronotyp relativ später ist. Gradisar et al. diskutieren, dass dies in Kombination mit frühem Schul- oder Ausbildungsbeginn dazu führen könnte, dass Jugendliche versuchen, durch frühere Bettgehzeiten (als physiologisch) dennoch eine möglichst angemessene Schlafdauer zu erreichen. Dies könnte zu langen Einschlaflatenzen führen, die wiederum Grübeln begünstigen und so das Risiko für die Entstehung von Depressionen erhöhen [1].

### Gesundheitsrisiko Schlafstörungen.

Zusammenfassend liegen für die gesundheitlichen Folgen von Schlafstörungen sowohl experimentelle Studien als auch epidemiologisch-klinische Studien zu Schlafmangel (verminderter Nachtschlaf) oder Insomnie (Einschlaf‑, Durchschlafstörungen, verminderte Schlafqualität) vor, die in Metaanalysen auf vielfältige und negative gesundheitliche Konsequenzen bei Erwachsenen hinweisen: Bezüglich der Kognition sind negative Effekte auf die Gesamtperformanz, Exekutivfunktion, Daueraufmerksamkeit und Langzeitgedächtnis festzustellen [15]. Eine aktuelle Metaanalyse zeigt [16], dass es bei Schlafverlust zu einer moderaten Erhöhung der negativen Stimmung kommt. Hierbei zeigte sich ein umso stärkerer negativer Effekt von Schlafverlust auf die Stimmung, je jünger die Proband:innen waren.

Die erste Metaanalyse zu Schlaf, Kognition und Verhaltensproblemen [17] bei Kindern im Schulalter (5–12 Jahre) berichtet für epidemiologische Stichproben übereinstimmend einen positiven Zusammenhang zwischen Schlafdauer und kognitiver Leistung sowie Exekutivfunktion, jedoch nicht mit der Daueraufmerksamkeit. Dieses leicht abweichende Ergebnis im Vergleich zu den Studien bei Erwachsenen wird von den Autor:innen auf methodische Probleme und Unterschiede in der Gehirnentwicklung zurückgeführt. Kürzerer Schlaf hängt bei Kindern gemäß der Metaanalyse ebenfalls mit gleichermaßen externalisierenden wie internalisierenden Verhaltensstörungen zusammen.

### Schlaf und Depression.

In adulten Stichproben zeigen nahezu dreiviertel der Proband:innen mit einer diagnostizierten Depression Einschlafinsomnie, Durchschlafinsomnie und frühmorgendliches Erwachen [18]. Neben der bereits genannten hohen Komorbidität von Depressionen und Schlafstörungen im Jugendalter (71 %; [5]) zeigten auch populationsbasierte Schätzungen bei Jugendlichen in einer großen Studie in Großbritannien [19] bei 77 % der 16- bis 24-Jährigen ein gemeinsames Auftreten von depressiver Symptomatik (ermittelt durch klinisches Interview nach ICD-10-Kriterien) und mindestens einem Insomniesymptom (einzelne Symptome ermittelt durch klinisches Interview, ohne zwingend erforderliche Erfüllung einer vollständigen Diagnose). Die Autor:innen argumentieren, dass Insomnie als Screeninghilfe für Depression dienen könnte, da in der Gesamtstichprobe 96 % der Teilnehmenden, die nicht von Depressionen betroffen waren, auch keine Insomniesymptome berichteten (doppelt negativ), während 41 % der von Depressionen betroffenen Teilnehmenden, auch eine vollständige Insomniediagnose erfüllten (doppelt positiv). Die Autor:innen folgern daraus, dass das Diagnostizieren einer Depression *ohne* Schlafprobleme besonderer Abwägung bedarf.

Hypersomnie ist insgesamt seltener und eher mit atypischer Depression assoziiert. Sie tritt jedoch häufiger beim weiblichen Geschlecht und viermal so oft bei jüngeren Patient:innen (unter 30 J.) im Vergleich zu älteren auf [20]. Daher könnte diese Symptomatik von besonderem Interesse in einer jungen Kohorte sein. Ergebnisse zeigen, dass sowohl eine unterdurchschnittliche als auch eine überdurchschnittliche Schlafdauer mit affektiven Symptomen bis hin zu mehr Suizidalität einhergeht [21].

Ausgeprägte Schlafstörungen sind mit einem erhöhten Risiko für Suizidgedanken, -versuche und Suizid verbunden, auch unabhängig von Depressionen [22]. Gestörter Schlaf und Depression treten häufig gemeinsam auf: Eine prospektive Längsschnittstudie, durchgeführt mit Adoleszenten in Australien, zeigte, dass depressive Symptome prädiktiv für später auftretende Schlafstörungen sind [23]. Hierzu gehören eine erhöhte Einschlaflatenz, ein erhöhter Anteil von Wachheit während der Schlafzeit und eine verkürzte Schlafzeit. Da Jugendliche mit einer Depression sich oft zurückziehen, ihre Aktivitäten einschränken, auch tagsüber nicht selten im Bett liegen und sich bei ihnen wiederholende, quälende Gedanken häufig aufdrängen, könnten es diese Verhaltensänderungen sein, die in Verbindung mit den kognitiven Störungen zu vermehrter Schlaflosigkeit führen [14]. Das nächtliche Gedankenkreisen wiederum verstärkt die Schlaflosigkeit und der Gedanke an das Zubettgehen in Verbindung mit der Angst vor der Schlafstörung verstärkt wiederum die Einschlaflatenz. Es liegt Evidenz für einen bidirektionalen Zusammenhang zwischen Depression und Schlafstörung vor, wie er auch in weiteren Längsschnittstudien beschrieben wurde [24]. Daher scheint es durchaus relevant, Schlafstörungen als Risiko für eine Depression im Jugendalter zu betrachten und präventiv möglichst frühzeitig Schlafstörungen im Jugendalter zu vermeiden und zu reduzieren.

## Chronobiologische und schlafmedizinische Diagnostik

### Anamnese

Innerhalb der klinischen Anamneseerhebung sollten Schlafstörungen und circadiane Besonderheiten standardmäßig erfasst werden. Weiterführende Diagnostikverfahren dienen dazu, die Behandlung bei Heranwachsenden an chronobiologischen und schlafmedizinischen Erkenntnissen zu orientieren. Die Verfahren dienen einerseits zur komplementären Diagnostik bei Depression mit einhergehenden Schlafstörungen. Andererseits können diese Verfahren auch diagnoseübergreifend zur genaueren Eruierung vorliegender circadianer Unregelmäßigkeiten/Schlafstörungen dienen. Sollte der Verdacht einer organischen Genese bestehen, ist diese zunächst abzuklären und entsprechend zu behandeln.

### Chronobiologische Messmethoden

#### Fragebögen.

Im klinischen Alltag sind aktuell Fragebogenverfahren empfehlenswert. In Tab. 1 sind die im Folgenden genannten Verfahren zusammengefasst. Zur Erfassung des Chronotypen stehen im Wesentlichen zwei etablierte Fragebogenverfahren zur Verfügung. Das entscheidende Unterscheidungsmerkmal ist das zugrunde liegende Konzept des Chronotypen. Der sogenannte Morningness-Eveningness-Fragebogen (MEQ) evaluiert den Chronotyp im Sinne eines psychologischen Konstruktes. Hierzu werden die tageszeitlichen Präferenzen für bestimmte Aktivitäten erhoben. Anhand einer Summenpunktezahl erfolgt schließlich die Einteilung in „Früh-Typen“, „Intermediäre Typen“ und „Abend-Typen“. Für Kinder und Jugendliche adaptierte und validierte Versionen, der Morningness Eveningness Questionnaire for Children and Adolescents (MEQ-CA) sowie Morningness-Eveningness Scale for Children (MESC), liegen vor. Der Munich ChronoType Questionnaire (MCTQ) hingegen evaluiert den Chronotyp als biologisches Konstrukt. Hierbei spielen die quantifizierbaren Zeitpunkte für Schlaf und Wachheit die zentrale Rolle. Sie werden getrennt für freie und Arbeits‑/Schultage erhoben. Schlafzeitpunkte an freien Tagen werden verwendet, da davon ausgegangen wird, dass freie Tage repräsentativer für die Synchronisierungsphase sind, nachdem weniger äußere Faktoren das Schlaf-Wach-Verhalten beeinflussen. Durch die Berechnung der Variablen separat an freien und an Arbeits‑/Schultagen ist die Feststellung von sozialem Jetlag möglich. Der Fragebogen ist online verfügbar.^1^ Ein validierter Fremdreport für 4‑ bis 11-jährige Kinder (Children’s Chronotype Questionnaire, CCTQ), entwickelt basierend auf dem MCTQ, liegt ebenfalls vor (online verfügbar mit Erlaubnis der Autor:innen)^2^.

**Table 1:** 

Verfahren	Messgegenstand	Name/Modalität	Validierung/Anwendung	Referenzen
Fragebogen	Chronotypisierung	Morningness-Eveningness-Questionnaire (MEQ)	Erwachsene	Dt. Übersetzung: Griefahn B, Künemund C, Bröde P, Mehnert P. [53]
		Munich ChronoType Questionnaire (MCTQ)	Erwachsene	Roenneberg T, Wirz-Justice A, Merrow M. [54]
		Children’s Chronotype Questionnaire (CCTQ)	4–11 Jahre	Werner H, LeBourgeois MK, Geiger A, Jenni OG. [55]
		Morning-Eveningness-Scale for Children (MESC)	11–18 Jahre	Carskadon MA, Vieira C, Acebo C. [56]
		MEQ for Children and Adolescents (MEQ-CA)	12–20 Jahre	Tonetti L, Adan A, Di Milia L, Randler C, Natale V. [57]
	Tagesschläfrigkeit	Epworth Sleepiness Scale (ESS)	Erwachsene	Dt. Übersetzung: Bloch KE, Schoch OD, Zhang JN, Russi EW. [58]
		ESS for Children and Adolescents (ESS-CHAD)	12–18 Jahre	Janssen KC, Phillipson S, O’Connor J, Johns MW. [59]
	Schlafqualität	Pittsburgh Sleep Quality Index (PSQI)	14–24 Jahre und Erwachsene	Dt. Übersetzung: Hinz A, Glaesmer H, Brähler E, Löffler M, Engel C, Enzenbach C, Hegerl U, Sander C. [60]
	Insomnie-Schweregrad	Insomnia Severity Index (ISI)	12–19 Jahre und Erwachsene	Bastien CH, Vallières A, Morin CM. [61]
		Sleep Disorders Inventory for Students-Children (SDIS-C)	2–18 Jahre	Luginbuehl M, Bradley-Klug KL, Ferron J, Anderson WM, Benbadis SR. [62]
		Sleep Disturbance Scale for Children (SDSC)	6–15 Jahre	Bruni O, Ottaviano S, Guidetti V, Romoli M, Innocenzi M, Cortesi F, Giannotti F. [63]
Aktimetrie	Chronotypisierung, Schlaf-Wach-Verhalten	Aktimeter am Handgelenk getragen	Erwachsene und Kinder	Roenneberg T, Keller LK, Fischer D, Matera JL, Vetter C, Winnebeck EC. [64]
DLMO (Dim Light Melatonin Onset)	Chronotypisierung	Speichel, Urin, Blut	Erwachsene und Kinder	De Almeida EA, Di Mascio P, Harumi T, Spence DW, Moscovitch A, Hardeland R, Cardinali DP, Brown GM, Pandi-Perumal SR [65]. Wittenbrink N, et al. [28]
Polysomnographie	Schlafstörungen	Verschiedene physiologische Messmethoden (z. B. EEG, EKG), optional zusätzlich Video‑/Tonaufnahmen	Erwachsene und Kinder	Augustinavicius JL, Zanjani A, Zakzanis KK, Shapiro CM. [66]

#### Melatoninmessung.

Melatonin ist ein zentralnervös (in der Epiphyse) hergestelltes Hormon, dass enzymatisch aus Serotonin gebildet wird, die Blut-Hirn-Schranke gut passiert und sowohl im Gehirn (Hippocampus, Amygdala) als auch peripher wirksam ist. Melatonin hat neben schlafanstoßenden auch antioxidative und antiinflammatorische Wirkungen. Der Melatoninspiegel steigt bei gesunden Menschen ca. 1–3 h vor dem Einschlafen an und fällt innerhalb von ca. einer Stunde nach dem Aufwachen wieder ab. Die Ausschüttung ist an die Lichtverhältnisse (auch von Raumlicht im Haus) gekoppelt (Ausschüttung bei Dunkelheit, Abbau bei Helligkeit) und assoziiert mit zunehmender Schläfrigkeit sowie sinkender Körperkerntemperatur [25].

Die Erstellung eines zeitlichen Profils der Melatoninausschüttung stellt die Goldstandardmethode zur Erfassung der Phasen der circadianen Uhr dar [26]. Der DLMO (*Dim Light Melatonin Onset*) markiert dabei den Zeitpunkt, an dem die natürliche Tages-Melatonin-Ausschüttung einen Schwellenwert von 10 pg/ml im Plasma (DMLO10) oder 3 pg/ml im Speichel (DMLO3) überschreitet, wodurch die schlafanstoßende Wirkung ausgelöst wird.

Die Messung der Melatoninausschüttung wird aktuell vorwiegend zu wissenschaftlichen Zwecken durchgeführt und Ausschüttungsprofile können im Speichel, Blutserum oder Urin bestimmt werden [26]. Hierbei müssen (bisher noch) mehrere abendliche Proben unter standardisierten Bedingungen genommen werden (z. B. Tragen von Brillen, die blauwelliges Licht filtern/Einhaltung bestimmter Zeitabstände; [27]). In Anbetracht des hohen Umsetzungsaufwands ist die Anwendbarkeit im klinischen Alltag eingeschränkt. Zukünftig wäre eine einfach umsetzbare und kostengünstige Phasenbestimmung mit wenigen Messzeitpunkten wünschenswert, um beispielsweise den optimalen Zeitpunkt der Melatoningabe festlegen zu können. Dies ist Gegenstand aktueller Forschung [28].

#### Aktimetrie.

Die kontinuierliche Erfassung lokomotorischer Aktivität über einen Zeitraum mehrerer Tage bis Wochen gibt Aufschluss über tägliche Aktivität und Ruhe bzw. Schlaf und Wachheit sowie deren zeitliche Verteilung und Muster. So lassen sich beispielsweise Unregelmäßigkeiten oder Veränderungen des Schlafes feststellen. Lokomotorische Aktivität kann mit dem sogenannten Aktimeter erfasst werden (wie eine Smartwatch am Handgelenk getragen). Das Aktimeter erfasst Bewegungsereignisse durch ein integriertes Akzelerometer. Mittels spezialisierter Software erfolgt die Auswertung der erhobenen Messungen und es ist beispielsweise möglich, den sog. Aktivitätsschwerpunkt zu berechnen (maximaler Punkt der Aktivität entlang einer angepassten 24-Stunden-Cosinuskurve). Dieser „Aktivitätsschwerpunkt“ kann als Näherungsvariable für den Chronotyp verwendet werden, da gezeigt werden konnte, dass er mit anderen Messmethoden gut korreliert [29]. Auch hier bestehen Herausforderungen bei der klinischen Anwendung und der Einsatz findet aktuell zumeist im wissenschaftlichen Kontext statt: Das Aktimeter sollte über einige Tage kontinuierlich (auch nachts) getragen werden und die generierten Daten müssen mittels spezieller Software ausgewertet werden.

### Schlafmedizinische Diagnostik

#### Fragebögen und Schlaftagebuch.

Zur Diagnostik des Schlafes stehen verschiedene Fragebögen zur Verfügung (Tab. 1). Auch wird das Führen eines einfachen Schlaftagebuchs empfohlen. Bei jüngeren Kindern erfolgt die Erhebung fremdanamnestisch durch die Eltern, wobei zu beachten ist, dass Eltern die Schlafdauer tendenziell überschätzen und die Häufigkeit nächtlichen Erwachens unterschätzen. Insgesamt zeigen Schlaftagebücher jedoch eine gute Übereinstimmung mit objektiven Messungen (z. B. Aktimetrie; [30]) und eine Kombination mit Aktimetrie kann je nach klinischer Fragestellung erwogen werden. Weiterhin kann der Insomnia Severity Index (ISI) zur Erfassung des Insomnie-Schweregrads im Jugendalter eingesetzt werden. Der im Erwachsenenbereich gängige Pittsburgh Sleep Quality Index (PSQI) ist in wenigen Studien auch bei Heranwachsenden eingesetzt worden und zeigte eine gute Validität und Reliabilität (nicht in deutscher Sprache getestet; [31]).

In einer Übersicht über Kinderschlaffragebögen folgern die Autor:innen, dass das Sleep Disorders Inventory for Students–Children (SDIS‑C; [32]) und die Sleep Disturbance Scale for Children (SDSC; [33]) alle von den Autor:innen festgesetzten Gütekriterien erfüllen (u. a. Reliabilität und Validität), wobei diese Verfahren noch nicht ins Deutsche übersetzt wurden [32]. Eine speziell für Kinder und Jugendliche validierte Version der Epworth-Schläfrigkeitsskala für Tagesschläfrigkeit (ESS), der ESS-CHAD, existiert und ist online abrufbar.^3^ Er misst die Tagessschläfrigkeit in verschiedenen Alltagssituationen.

#### Polysomnographie.

Zur genaueren Diagnostik von Schlafstörungen kommt die Polysomnographie zum Einsatz (Tab. 1). Sie umfasst neben der Ableitung der Hirnströme (Elektroenzephalographie, EEG) und der Ableitung der Herzaktion (Elektrokardiographie) auch die Messung verschiedener Vitalparameter sowie optional Video‑/Tonaufnahmen. Sie sollte bei begründetem Verdacht zum Ausschluss organischer Schlafstörungen verwendet werden. Bei Heranwachsenden zeigt die Polysomnographie weniger klare Unterschiede zwischen Depression und gesundem Zustand. Die Mehrheit der Studien zeigt keine signifikanten Unterschiede, während einzelne Studien Veränderungen in der Schlafarchitektur und beispielsweise mehr *Rapid-eye-movement*-Schlafphasen feststellen [14]. Auch wurden EEG-Muster, sowohl bei Erwachsenen als auch bei Jugendlichen, bereits auf ihre Vorhersagekraft für Behandlungsansprechen, Prognose und Rezidiv untersucht. Jedoch spielen Geschlecht und Alter für die Schlafarchitektur eine große Rolle und lassen noch keine klare Schlussfolgerung zu [33].

## Chronobiologische und schlafmedizinische Behandlungsansätze

### Melatoningabe.

Im Kindes- und Jugendalter zeigt sich aktuell eine heterogene Datenlage zur Effektivität von Melatonin bei der Behandlung von Schlafstörungen bei verschiedenen psychischen Erkrankungen (Aufmerksamkeitsdefizit-Hyperaktivitätsstörung, Autismusspektrumstörung, Depression; [34]). Melatonin wird im Kindes- und Jugendalter in Dosierungen von 2–5 mg verabreicht, meist 1–2 h oder kürzer vor dem Schlafengehen. Die Halbwertzeit oral verabreichten Melatonins bei Kindern liegt zwischen 30–45 min, die Bioverfügbarkeit schwankt zwischen 1–37 % [35]. Zu den gelegentlichen Nebenwirkungen gehören Kopfschmerzen, Schwindel, Durchfall, Hautausschlag und Benommenheit. In einigen Studien konnten verkürzte Schlaflatenzen, verbesserte Schlafdauer und weniger Durchschlafstörungen festgestellt werden. Bei einer Gabe von 3–6 mg Melatonin vor dem Einschlafen wurde eine verkürzte Einschlafzeit im Durchschnitt von 0,6 h und eine verlängerte Schlafzeit von einer halben Stunde gemessen (systematischer Review, [36]), außerdem eine begünstigte frühere bzw. verbesserte Synchronisierung von Innen- und Außenzeit, die zur Reduktion von sozialem Jetlag beitragen kann [37]. Da kein bedeutsamer Zusammenhang zwischen der Dosis des verabreichten Melatonins und der Wirksamkeit auf den Schlaf gefunden wurde, sollte bei einer Behandlung bei Kindern und Jugendlichen mit einer niedrigen Dosierung begonnen werden (z. B. 1 mg). Empfohlen wird eine Gabe zwischen 2–5 h vor DLMO [36].

Andererseits gibt es Untersuchungen, die keine signifikanten Auswirkungen einer Melatoningabe auf Einschlafdauer, Gesamtschlafdauer oder depressive Symptome zeigen konnten [38]. Eingang in die aktuellen Behandlungsempfehlungen von Schlafstörungen hat bisher die Behandlung mit Melatonin bei Kindern und Jugendlichen mit Autismusspektrumstörungen und Aufmerksamkeitsdefizit-Hyperaktivitätsstörung, unterstützt von Umgebungs- und Verhaltensmaßnahmen, erhalten [39].

### Lichttherapie.

Insbesondere für Erwachsene zeigt die Lichttherapie gute Evidenz in der Behandlung von Depressionen. Typischerweise wird eine Lichtexposition nach dem Aufstehen für ca. 30 min mit 10.000 lx starkem Licht empfohlen. Als Monotherapeutikum und auch als adjuvante Therapie (z. B. zusätzlich zu Psychotherapie, Medikation, Morgensport) zeigt Lichttherapie gute Erfolge bei saisonalen und nicht-saisonalen Depressionen und kann auch zur früheren Synchronisierung von Innen- und Außenzeit (ggf. Verminderung des sozialen Jetlags) eingesetzt werden [40, 41].

Im Kindes- und Jugendalter sind diese Resultate bisher nicht eindeutig [1]. Eine randomisierte kontrollierte Studie [42] zeigte eine signifikante Reduktion der Depressionssymptomatik im stationären kinder- und jugendpsychiatrischen Setting bei Jugendlichen mit einer Depression nach einer zusätzlichen Behandlung mit Lichttherapie (10 Sitzungen, 10.000 lx, Dauer 45 min morgens zwischen 08–10 Uhr). Trotz der noch unzureichenden Studienlage könnte eine Lichttherapie zusätzlich zur Psychotherapie und/oder Pharmakotherapie bei Depression im Jugendalter zu Verbesserungen der Einschlaflatenz und Rückgang der depressiven Symptomatik sowie zur Verbesserung des Funktionsniveaus beitragen [43].

### Schlafhygiene und verhaltenstherapeutische Ansätze.

Für den Ansatz der Schlafdeprivation bei Kindern und Jugendlichen mit einer Depression und Insomnie existieren nur wenige Einzelstudien, sodass die Evidenz nicht ausreichend ist [44]. Dagegen ist die Rolle von Schlafhygiene mit geeigneter Schlafumgebung (z. B. keine Uhr am Bett, ruhige Umgebung, wenig Lichteinfall), entspannenden Einschlafritualen und einem Verzicht auf abendlichen Bildschirmkonsum von besonderer Bedeutung [45] – auch in Anbetracht einer eher unsicheren Datenlage zur Pharmakotherapie bei Schlafstörungen im Kindes- und Jugendalter [46]. Weitere Maßnahmen der Schlafhygiene sind u. a., dass das Bett lediglich zum Schlafen aufgesucht werden sollte; Tagschlaf sollte eher vermieden bzw. kurzgehalten werden (ca. 10–20 min, nicht mehr ab dem späten Nachmittag); Lichtexposition in der ersten Tageshälfte ist förderlich für ein regelmäßiges Schlaf-Wach-Verhalten (regelmäßige Schlafenszeiten); sollte das Einschlafen nicht möglich sein, sollte das Bett nach ca. 15 min verlassen werden und kurzzeitig einer ruhigen Aktivität nachgegangen werden. Für Jugendliche ab 13 Jahren wird eine Schlafdauer von 9–9,2 h als optimal erachtet [1]. Kognitive Verhaltenstherapie (KVT) mit Insomniefokus (Cognitive Behavioral Therapy for Insomnia, CBT-I) ist bei Heranwachsenden insgesamt effektiv und anhaltend (untersuchte Effekte bis zu 12 Monate nach Intervention u. a. auf Insomnie-Schweregrad, Einschlaflatenz, Schlafeffizienz; [47]), so zeigte diese Metaanalyse über verhaltenstherapeutische Schlafinterventionen signifikante Verbesserungen der Gesamtschlafzeit sowie der Einschlaflatenz. CBT‑I beinhaltet meist psychoedukative Elemente (über Schlaf und dessen Störungen), Einheiten zur Stimuluskontrolle, Schlafhygiene und Entspannungsverfahren. Das deutschsprachige JuSt-Manual von Schlarb et al. ist hierbei als bisher vielversprechend untersuchtes Therapieprogramm für Jugendliche ab 11 Jahren zu nennen [48]. Die KVT-basierte Intervention wird auch digital (dCBT-I) angeboten [49], auch wenn die persönliche Einbindung der Therapeut:innen meist zu höheren Behandlungseffekten führt [50].

## Empfehlungen

Die Berücksichtigung der circadianen Rhythmik, des Schlaf-Wach-Verhaltens und die Diagnostik von Störungen des Schlafes sind bei Depressionen im Jugendalter, in Anbetracht der zusammengefassten Evidenz, von besonderer Bedeutung. Daher sollten Beeinträchtigungen des Schlafes standardmäßig in der Anamnese, der klinischen Diagnostik und Behandlungsplanung bei Depressionen erfragt werden. Neben der Anamneseerhebung stehen diagnostisch insbesondere Fragebögen und Schlaftagebücher niedrigschwellig im klinischen Alltag zur Verfügung. Die Reduktion von sozialem Jetlag sollte angestrebt werden. Hierzu sind, neben individuellen Maßnahmen, auch Veränderungen auf gesellschaftlicher Ebene wichtig: Ein flexiblerer Schulbeginn für Jugendliche ist zu empfehlen, da Verbesserungen des Schlafes und der kognitiven Fertigkeiten in Modellversuchen gezeigt wurden [51]. Aufklärung über die Zusammenhänge von Schlafstörungen und Depressionen sollte Bestandteil der Versorgung sein. Maßnahmen zur Schlafhygiene sollten, auch im Hinblick auf Prävention und die Reduktion des Wiedererkrankungsrisikos, mit den Kindern bzw. Jugendlichen und deren Familien besprochen und gefördert werden. In der klinischen Versorgung werden häufig neben Melatonin, Neuroleptika wie Quetiapin, Antidepressiva und Benzodiazepine zur Verbesserung des Schlafes bei Kindern und Jugendlichen eingesetzt [52], die nicht selten mit unerwünschten Nebenwirkungen einhergehen. Daher sollten primär Maßnahmen wie Psychoedukation, Schlafhygiene und umweltbezogene Maßnahmen bei Kindern und Jugendlichen eingesetzt werden. Die weiteren genannten Behandlungsmaßnahmen (Lichttherapie, Gabe von Melatonin) können bspw. als adjuvante Therapeutika in der Behandlung von Depressionen eingesetzt werden. Für den wissenschaftlichen Bereich besteht Bedarf nach validierten Messinstrumenten für das Jugendalter (z. B. zur Chronotypisierung/spezifischen Feststellung von Schlafstörungen). Ebenso fehlt es an hochwertigen kontrollierten Studien zu den verschiedenen Interventionen zur Verbesserung des Schlafes für Jugendliche mit psychischen Erkrankungen.

## Fazit

Chronobiologische und schlafmedizinische Aspekte sind hinsichtlich Depressionen im Kindes- und Jugendalter von hoher Relevanz. Zusammenhänge zwischen gestörter circadianer Rhythmik sowie gestörtem Schlaf und dem Vorliegen affektiver Erkrankungen sind in der Forschungsliteratur gut charakterisiert. Eine stärkere Berücksichtigung dieser Aspekte sollte in der klinischen Versorgung erfolgen. Hierzu sind eine symptomspezifische Anamneseerhebung, medizinische Aufklärung, der Einsatz niedrigschwelliger Messinstrumente, Förderung der Schlafhygiene und Reduktion des sozialen Jetlags empfehlenswert. Es besteht der Bedarf, für die besondere Entwicklungsphase der Adoleszenz geeignete Messinstrumente und Interventionen an der Schnittstelle von Depressionen, Chronobiologie und Schlafmedizin zu entwickeln, um adoleszente Patient:innen mit Depressionen umfassender versorgen zu können.

## Footnotes

1 https://www.thewep.org/documentations/mctq Zugegriffen: 07.12.2023.

2 https://www.tandfonline.com/doi/full/10.1080/07420520903044505 Zugegriffen: 07.12.2023.

3 https://www.narcolepsylink.com/pediatric/recognize/epworth-sleepiness-scale-for-children-and-adolescents/ Zugegriffen: 07.12.2023.
